# Clustering-Conditioned Granger Causality Between GDP Growth and Private Financing

**DOI:** 10.3390/e28050510

**Published:** 2026-05-01

**Authors:** Roberto Flores-Nava, Edgar Roman-Rangel

**Affiliations:** Department of Computer Science, ITAM, Mexico City 01080, Mexico; rflore53@itam.mx

**Keywords:** finance–growth nexus, credit to private sector, GDP growth, Granger causality, clustering, XGBoost

## Abstract

Whether finance leads growth or growth leads finance remains a century-long debate. We argue that the direction and strength of the GDP–credit nexus are context-dependent and can be systematically uncovered by conditioning causal analysis on macro-structural heterogeneity across countries. We implement a two-stage pipeline: (i) unsupervised clustering of 30 economies (2005–2022, five annual macro indicators) via Agglomerative Clustering to form homogeneous macro-structural groups; and (ii) within-cluster dynamic causal analysis using lagged correlations, Granger causality and explanatory models (quarterly GDP and private credit, year-on-year growth, 1Q2005–3Q2024). Results show non-universality of causality: (a) in “developed and in transition, economically stable” economies, credit → GDP is predominant; (b) in “highly developed, competitive and stable” economies, bidirectionality is predominant; however, the results are not economically intuitive; (c) in “emerging/intermediate with macro risk”, bidirectional links are common, and feedback between both variables is interpretable across distinct scenarios. Post hoc Lasso and XGBoost confirm effect magnitudes and non-linear thresholds. We contribute a macro-segmented causal discovery framework that reconciles conflicting findings in the literature and provides policy-relevant differentiation by economic context.

## 1. Introduction

The relationship between financial development and economic growth has been debated for more than a century. This started with early theoretical perspectives that framed the central discussion: Schumpeter [1] proposed that financial institutions play a crucial role by channeling resources into innovative projects (i.e., by financing projects), which in turn drive long-term economic growth; while Robinson [2] argued that it is the dynamic economic environment what drives the conditions for financial development, suggesting the causal direction runs the other way around. This fundamental question—whether finance leads growth or growth leads finance—remains unresolved.

Subsequent empirical research documented systematic associations between financial depth and economic performance. King and Levine [3] and Levine [4] showed that financial development is strongly linked to long-run growth, while further evidence indicates that private credit is positively associated with productivity and GDP dynamics [5]. However, findings vary across countries, time horizons, and empirical specifications. Much of the literature relies on pooled panel models in which structural differences across economies are only partially controlled for, making results potentially sample-dependent.

This raises a deeper question: is the finance–growth nexus universal, or does it depend on the structural characteristics of each economy? More recent contributions emphasize heterogeneity and dynamic specification, showing that the direction and strength of causality may differ across countries and horizons [6,7,8].

Building on this perspective, this study proposes that the relationship between private credit and economic growth is context-dependent; rather than assuming a single global mechanism, we argue that the direction, magnitude, and functional form of the relationship may vary systematically according to macroeconomic structure. Countries differ in macroeconomic conditions such as labor market conditions, financial depth, inflation and growth dynamics; these structural dimensions may condition how credit and output interact over time.

To test this hypothesis, we adopt a two-stage empirical strategy. First, countries are segmented into structurally homogeneous groups using unsupervised learning by applying clustering techniques based on annual macroeconomic indicators. Second, within each group, we evaluate the dynamic relationship between private sector credit and GDP using correlation analysis and Granger causality tests. Finally, flexible non-linear explanatory models are estimated to characterize the magnitude and shape of the dynamic effects, moving beyond purely linear specifications.

By explicitly separating structural heterogeneity from dynamic interactions, this approach allows us to determine whether the finance–growth nexus follows distinct patterns across types of economies. The contribution of this study is therefore both methodological and empirical: it integrates macroeconomic segmentation with causal testing and non-linear modeling to provide a more granular view of the finance–growth relationship. The results suggest that the direction and functional form of the link between credit and growth depend on the economic context, challenging the notion of a single universal mechanism.

The remainder of this paper is organized as follows. Section 2 presents related works. Section 3 describes the data sources, description, representativeness, transformation, and quality for the statistical techniques. Section 4 highlights the methodology followed and the techniques employed. Section 5 shows the results and their interpretation. Section 6 presents a discussion of our findings. Finally, Section 7 refers to the conclusion of this analysis.

## 2. Related Work

The question of whether finance leads or follows economic growth has been debated for more than a century. Schumpeter [1] viewed financial systems as drivers of innovation and economic development, while Robinson [2] argued that finance largely responds to growth rather than causing it.

Empirical work later attempted to quantify this relationship more systematically. To mention some, Goldsmith [9] documented that countries with deeper financial systems tended to grow faster. McKinnon and Shaw [10,11] further argued that financial repression may constrain investment and that liberalization could instead stimulate economic activity.

Some decades later, in the 1990s, King and Levine [3] showed that the financial depth is strongly associated with long-run growth. Levine [4] reviewed the literature and outlined the main channels linking finance and growth, helping to consolidate the previous results. Then, Beck, Levine, and Loayza [5] also showed that private credit matters for productivity and GDP, while recognizing at the same time that causality could go both ways.

More recent contributions have shifted the focus toward heterogeneity. Mhadhbi et al. [6] applied bootstrap panel Granger tests and found that causal direction varies across countries. Abbas et al. [7] introduced a time–frequency approach, showing that results may differ between short- and long-run horizons. Similarly, Kchikeche and Mafamane [8] documented horizon-dependent causality patterns in a country-level study.

Mahlangu and Mongale [12] investigate the relationship between financial development and economic growth in South Africa. The study relies on annual time series from 1980 to 2022 from the South African Reserve Bank and Quantec Easy Data. Although that work evaluates causality in terms of the Granger approach, it does it by comparing GDP to interest rate liberalization, but not against the finance–growth nexus. Furthermore, they focus on South African economy, not needing to perform clustering of different countries.

The work of Fuinhas et al. [13] evaluates the Granger causality between economic growth, inflation, stock market development, and banking sector development for sixteen high-income countries, analyzing data from 2001 to 2016. They found evidence of bidirectional causality between all variables. However, their experiments neglect the need to group countries prior to causality analysis.

De Mier et al. [14] identify groups of countries with similar economic growth behaviors. First, they apply causal discovery techniques, and later, they perform a clustering of counties following a hierarchical progression over the predictors. They found that the associations between income or geographical location and the nature of economic growth processes may not always hold. Unlike our approach, that work first involves the causality test and only later involves the clustering step. Furthermore, it focuses on variables other than the finance–growth nexus and GDP.

Taken together, these studies highlight two important points. First, the finance–growth relationship is not universal. Second, results depend heavily on the empirical strategy and the context under consideration. However, most approaches either estimate pooled panels or allow heterogeneity implicitly through econometric techniques.

In contrast, this study incorporates heterogeneity directly into the empirical design. The countries are first segmented according to their macroeconomic structure, and then Granger causality tests are conducted within each group. Beyond identifying the predictive direction, explanatory models are subsequently estimated to characterize the magnitude and functional form of the relationship.

By separating structural differences across economies, and by combining causality testing with flexible modeling techniques, the analysis also contributes to the previous studies, with the result that the direction and shape of the finance–growth nexus is not a single global pattern: instead, it depends systematically on the type of economy.

**Figure 1:**
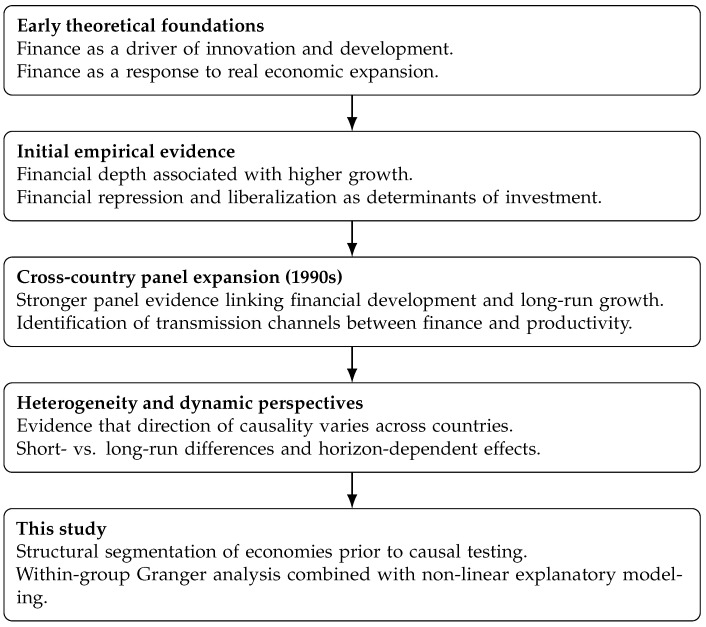
Evolution of the finance–growth literature and positioning of this study.

Figure 1 summarizes the main stages in the evolution of the finance–growth literature and illustrates how the present study builds upon and extends prior approaches.

## 3. Data

This study relies on three widely recognized international databases, combining annual and quarterly information to address two distinct analytical objectives: structural segmentation and dynamic causal analysis.

Quarterly GDP data was obtained from the IMF’s Quarterly National Accounts [15]. Quarterly private sector credit (as a percentage of GDP) was sourced from the Bank for International Settlements (BIS) [16]. Finally, annual macroeconomic indicators—including GDP growth, GDP per capita growth, inflation, unemployment, and private sector credit—were collected from the World Development Indicators (World Bank) [17].

The annual data was used for the clustering stage, as the objective in that step is to characterize long-run macroeconomic structure. In contrast, the quarterly data was used for causal analysis in order to capture short-term dynamic interactions and lag effects.

The clustering dataset covers the period 2005–2022. The quarterly dataset used for causal analysis spans from 1Q2005 to 3Q2024.

Using different frequencies reflects the distinct purposes of each stage: annual indicators capture structural economic profiles, whereas quarterly data allow the identification of temporal precedence and dynamic interactions.

### 3.1. Descriptive Statistics

The data is organized taking into account availability across the three sources and driven by three main criteria: (i) keep as many countries as possible with complete data; (ii) capture periods that are representative of different economics and financial conditions globally; and (iii) maintain a sufficient number of observations to apply statistical tests.

This analysis focuses on 30 countries: Australia, Austria, Belgium, Brazil, Chile, China, Colombia, Denmark, Finland, France, Germany, Greece, Hong Kong, Hungary, Ireland, Israel, Italy, Japan, Luxembourg, Malaysia, Mexico, The Netherlands, Norway, Poland, Portugal, South Africa, Spain, Sweden, United Kingdom, and United States of America. This dataset contains information from 2005 to 2022.

Five main macroeconomic variables were selected for the cluster analysis: GDP growth, GDP per capita growth, inflation, unemployment, and private sector credit (as a percentage of GDP). These variables were chosen because they jointly capture complementary dimensions of a country’s structural macroeconomic environment.

GDP growth reflects aggregate economic dynamism and short- to medium-term expansion capacity, while GDP per capita growth provides a development-adjusted measure that controls for demographic effects and allows for differentiation between population-driven and productivity-driven growth patterns. Inflation captures price stability and macroeconomic discipline, which are essential to understanding monetary conditions and economic credibility. Unemployment reflects labor market performance and structural rigidity, serving as a proxy for economic slack and social stability. Finally, private sector credit as a share of GDP measures financial depth and the relative size of the domestic financial system, providing a direct indicator of financing availability and leverage conditions.

Together, these variables allow for a multi-dimensional representation of macroeconomic structure, integrating growth dynamics, stability conditions, labor market performance, and financial development. This combination is particularly relevant for the hypothesis of the study, as it enables the identification of structurally homogeneous groups of countries before assessing whether the finance–growth nexus differs between economic contexts.

Table 1 reports the descriptive statistics for these main variables.

**Figure 2:**
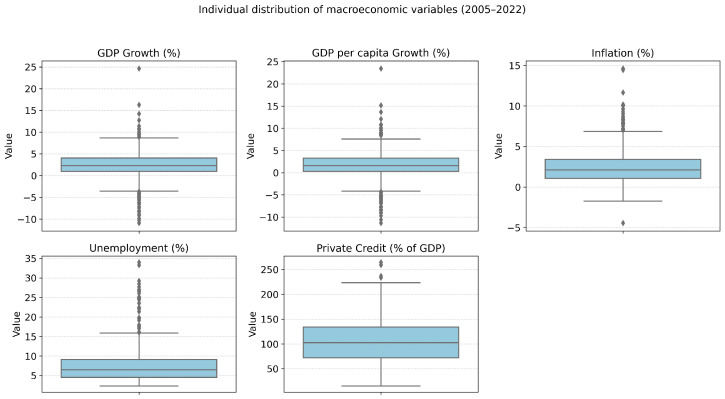
Distribution of macro economic variables used for cluster analysis (2005–2022).

In addition, in Figure 2, it is possible to note that the variables show a high heterogeneity; for example, the variables private credit and inflation show outliers and large dispersions, while GDP growth and per capita GDP growth exhibit less dispersion, despite still having a few outliers.

### 3.2. Preprocessing

The annual data from the World Bank used for the clustering were employed as published, without additional transformations.

For causal analysis, quarterly GDP and private sector credit were transformed to ensure comparability and suitability for dynamic modeling. Since the BIS credit series is expressed as a percentage of GDP, it was first converted into nominal credit levels in local currency using IMF quarterly GDP:

(1) $$C_{i,t}^{\mathrm{level}}=\left(\frac{C_{i,t}^{\%GDP}}{100}\right)\times GDP_{i,t},$$

where $C_{i,t}^{\%GDP}$ denotes private sector credit as a percentage of GDP for country *i* at quarter *t*, and $GDP_{i,t}$ represents nominal quarterly GDP in local currency units.

After obtaining credit in levels, both GDP and credit were transformed into year-on-year growth rates to ensure cross-country comparability, mitigate scale effects, and reduce seasonal distortions. The transformation was computed as

(2) $$\Delta X_{i,t}=\left(\frac{X_{i,t}-X_{i,t-4}}{X_{i,t-4}}\right)\times 100,$$

where $X_{i,t}$ corresponds to either nominal GDP or credit levels for country *i* at quarter *t*, and (t−4) captures the same quarter in the previous year. The use of year-on-year growth rates also helps approximate stationarity in the series, which is a standard requirement for Granger causality testing.

After computing year-on-year growth rates, the quarterly panel was reorganized to incorporate up to four quarterly lags of the relevant explanatory variable. For each country *i* and quarter *t*, the dataset contains the contemporaneous value of the target variable together with its corresponding lagged predictors, i.e., $X_{i,t-1},\ldots ,X_{i,t-4}$.

Only observations with complete contemporaneous and lagged information were retained, ensuring a consistent and balanced structure for the subsequent causal analysis.

### 3.3. Target Variables

For *cluster analysis*, the objective is to group countries into homogeneous clusters based on their structural macroeconomic profiles. This stage follows an unsupervised learning framework; therefore, no explicit target variable is defined. Instead, clustering relies on a criterion that maximizes intra-cluster homogeneity and inter-cluster heterogeneity.

Five annual indicators from the World Bank were selected as descriptive variables for this purpose: GDP growth, GDP per capita growth, inflation, unemployment, and private sector credit, as listed in Table 1. This results in a wide panel representation, where each row corresponds to a country and each column represents a specific year–variable combination from 2005 to 2022. Since five variables are observed over 18 years, the clustering matrix consists of 30 rows (countries) and 90 columns (five variables observed over 18 years).

In contrast, the *causal analysis* requires explicit dependent variables. The target variable $y_{i,t}$ corresponds to either the year-on-year growth rate of credit, denoted $\Delta C_{i,t}$, or the year-on-year growth rate of GDP, denoted $\Delta G_{i,t}$, for each country *i*, as defined in Section 3.

Two directional dynamic specifications were evaluated:

- Credit-led specification:
  (3) $$\Delta C_{i,t}=f\left(\Delta G_{i,t-1},\ldots ,\Delta G_{i,t-4}\right),$$
- Growth-led specification:
  (4) $$\Delta G_{i,t}=g\left(\Delta C_{i,t-1},\ldots ,\Delta C_{i,t-4}\right),$$

where f(·) and g(·) represent the functions associated with the distinct dynamic relationships in each direction; in other words, these ones represent that one variable might be in function of the other. In the first case, the objective is to evaluate whether past GDP growth helps explain credit growth; in the second, whether past credit growth helps explain GDP growth. This separation allows the dynamic structure and statistical significance of lagged effects to differ across directions.

This lag structure preserves temporal ordering and avoids contemporaneous simultaneity between growth and credit, enabling the evaluation of predictive dynamics consistent with the Granger framework.

For the causal stage, the quarterly panel of transformed growth rates was organized in longitudinal format and subsequently partitioned according to the structural segmentation obtained in the clustering stage. For each economic group, two directional datasets were constructed, resulting in six structured panels (three groups and two causal directions). These datasets constitute the empirical basis for correlation analysis, Granger causality testing, and subsequent explanatory modeling.

## 4. Methods

The methodological pipeline consists of five main steps: (i) data preparation, as already explained in Section 3, (ii) unsupervised learning for clustering of countries into homogeneous groups, (iii) correlation analysis between GDP and credit growth with quarterly lags; (iv) Granger causality tests to evaluate dynamic interactions; and (v) explanatory modeling to assess the magnitude and functional form of the dynamic effects.

**Figure 3:**
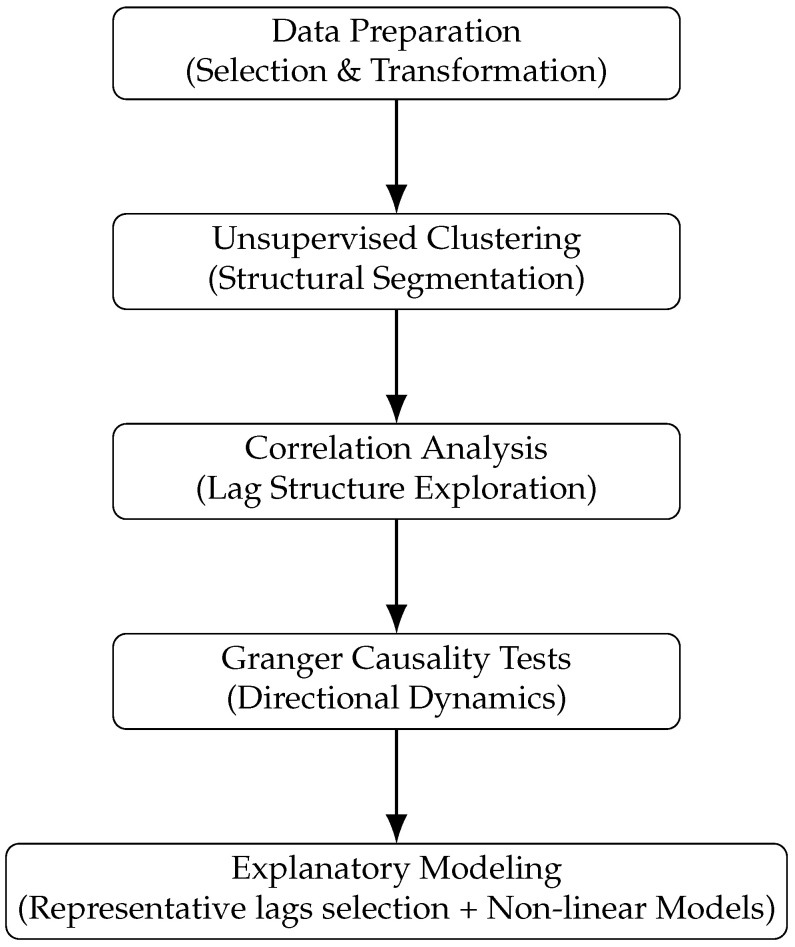
Methodological pipeline followed in this study.

Figure 3 illustrates the overall process. The following subsections describe steps (ii) through (v) in detail.

### 4.1. Cluster Analysis

Clustering algorithms were employed to group countries according to their structural macroeconomic characteristics. The objective of this stage was to identify homogeneous groups of economies sharing similar macroeconomic profiles prior to conducting the dynamic causal analysis.

Two techniques were tested: *k*-means and Agglomerative Clustering [18,19]. Model performance was assessed using the Silhouette Score [20], which measures the degree of cohesion within clusters and separation across clusters. In addition, dimensionality reduction techniques were used for visualization purposes to assess the stability and interpretability of the resulting partitions [21,22].

It is important to note that countries were not clustered based on raw time-series trajectories but on their annual macroeconomic indicators arranged as a wide panel, where each country is represented by a vector formed by five variables observed annually from 2005 to 2022, as explained in Section 3. Thus, each country is represented by a multi-dimensional feature vector. In this sense, the clustering stage reflects structural macroeconomic characteristics instead of short-term dynamics.

Since the objective was not to align time-series paths but to compare structural profiles, Dynamic Time Warping (DTW) was not required. The Euclidean distance was used as the dissimilarity metric. Alternative scaling procedures were explored during a preliminary analysis; however, the highest Silhouette Score was obtained using the variables on their original scale, so no standardization was applied in the final clustering specification.

Finally, boxplots of the five variables—GDP growth, GDP per capita growth, inflation, unemployment, and private credit—were analyzed to characterize each cluster, highlighting distinctive macroeconomic patterns (see Section 5.1).

### 4.2. Correlation Analysis

After clustering, pairwise correlations between GDP growth and credit growth were computed within each group of countries. The analysis considered both contemporaneous correlations and lagged relationships, incorporating up to four quarterly lags in each direction (see Section 5.2). That is, correlations were evaluated between $\Delta G_{i,t}$ and $\Delta C_{i,t-k}$, as well as between $\Delta C_{i,t}$ and $\Delta G_{i,t-k}$ for k=0,1,…,4, where $\Delta G_{i,t}$ and $\Delta C_{i,t}$ denote the year-on-year growth rates of GDP and credit, respectively, for country *i* at quarter *t*, where *k* represents the number of quarterly lags.

This step was intended as a preliminary assessment before conducting formal causality tests. By observing how correlations changed across lags, it was possible to detect potential lead–lag patterns between GDP growth and credit growth within each cluster.

Correlations were summarized as matrices and visualized using heatmaps for each group. This visualization facilitates comparison across clusters and highlights differences in the timing and magnitude of associations. The lag structure of correlations provides an intuitive indication of dynamic dependence that is later examined more formally through Granger causality tests.

Although correlation does not imply causality, this exploratory stage provides a sign of potential association between GDP growth and credit growth across clusters. In other words, the presence of lagged correlations suggests that dynamic interactions may exist; however, their functional form is not necessarily linear.

These results lead to two next steps: first, applying Granger causality tests to evaluate directional predictive relationships; and second, estimating non-linear models to capture effects that simple correlations or causality tests may not detect.

### 4.3. Granger Causality Tests

Correlation alone cannot establish directional relationships. Therefore, Granger causality tests [23] were performed to evaluate whether past values of one variable improve the prediction of the other.

Two competing models were estimated:

- Restricted model (autoregressive model without *X*):
  (5) $$Y_{t}=\alpha_{0}+\sum_{k=1}^{K}\alpha_{k}Y_{t-k}+u_{t}.$$
  This model uses only past values of the target variable, either GDP growth or credit growth.
- Unrestricted model (including an external variable *X*):
  (6) $$Y_{t}=\beta_{0}+\sum_{k=1}^{K}\beta_{k}Y_{t-k}+\sum_{k=1}^{K}\gamma_{k}X_{t-k}+\varepsilon_{t}.$$
  In addition to past values of the target variable, this model incorporates lagged values of the alternative variable (GDP growth or credit growth, depending on the direction tested).
  where
- $Y_{t}$: Target variable at time *t* (either $\Delta G_{t}$ or $\Delta C_{t}$);
- $X_{t-k}$: Lagged values of the alternative variable *X* at lag *k* (time t−k);
- $Y_{t-k}$: Variable *Y* at time t−k (lagged target variable);
- $\alpha_{0}$, $\beta_{0}$: Intercept terms;
- $\alpha_{n},\beta_{n},\gamma_{n}$: Slope coefficients;
- $u_{t}$, $\varepsilon_{t}$: Stochastic error terms;
- *K*: Number of lags considered in the specification.

The null hypothesis of no Granger causality from *X* to *Y* is

$$H_{0}:\gamma_{1}=\gamma_{2}=\cdots =\gamma_{N}=0,$$

where $\gamma_{n}$ denotes the coefficient associated with the *n*-th lag of the explanatory variable *X*. Under this null hypothesis, past values of *X* do not provide additional predictive power for *Y* beyond the information contained in its own lagged values. The alternative hypothesis states that at least one $\gamma_{n}\neq 0$.

The hypothesis is evaluated using an F-statistic that compares the restricted model in Equation (5) with the unrestricted model in Equation (6).

The procedure was applied in both directions (GDP → credit and credit → GDP) within each cluster, allowing the analysis to capture whether the dynamic relationship depends on the type of economy.

It is important to note that Granger causality evaluates whether past values of *X* improve the prediction of *Y* relative to a restricted model that includes only lagged values of *Y*. In our context, this corresponds to comparing the unrestricted specification in Equation (6) with the restricted model in Equation (5). Thus, the test captures predictive direction based on temporal precedence, but not structural or mechanistic causality.

### 4.4. Explanatory Models

The Granger causality tests provide evidence of predictive direction; however, they do not quantify the magnitude or functional form of the impact between variables. To further characterize these relationships, supervised learning algorithms were used as explanatory models, training them for each cluster. Notice that, at this point, these algorithms are used merely as means to quantify the contribution of each independent variable to the causality direction. However, they are not intended as predictors, and therefore we do not focus on potential overfitting scenarios.

As an initial benchmark, linear specifications were explored. In particular, Lasso regression [24] was used to identify the most relevant lagged terms of one variable in explaining the other. Given the potential multicollinearity across lagged regressors, Lasso serves as a regularization and variable-selection tool, shrinking less informative lags toward zero while retaining the most predictive ones. These selected lags were then used to inform subsequent modeling stages.

For the Lasso stage, the Elastic Net mixing parameter was fixed at α=0.9, favoring a penalization scheme close to Lasso. This choice was intentional, as the objective of this stage is not predictive optimization but the identification of a parsimonious and interpretable set of representative lags. In preliminary analyses, lower values of α resulted in the selection of a relatively large number of lagged predictors, which reduced interpretability and weakened the purpose of the preselection step. In contrast, α=1 (pure Lasso) showed to be very restrictive, consistently retaining only one lag across all the specifications. Therefore, α=0.9 was implemented to better balance sparsity and information retention. It is worth noting that α is a hyperparameter that can be further explored or tuned depending on the specific scenario.

The idea behind this choice was to make the selection stricter, so that only the lag(s) with meaningful explanatory power remain, and these can then be analyzed in more detail in the subsequent modeling stage.

However, the main focus of the analysis lies in capturing potential non-linear effects. For this purpose, the *Extreme Gradient Boosting* (XGBoost) algorithm [25] was employed for each cluster and for each direction of analysis.

XGBoost is based on gradient boosting of decision trees, where trees are added sequentially to correct the errors of previous iterations. In its general form, the prediction function can be written as

(7) $$F_{M}\left(x\right)=\sum_{m=1}^{M}\gamma_{m}h_{m}\left(x\right),$$

where $h_{m}\left(x\right)$ denotes the tree fitted at iteration *m*, $\gamma_{m}$ its associated weight, and *M* the total number of trees. This additive structure allows the model to capture interactions and non-linear relationships between lagged predictors and the target variable.

In this study, XGBoost was used primarily for interpretative purposes rather than pure prediction. Two tools were central in this stage. First, Partial Dependence Plots (PDPs) [26] were used to analyze how the predicted value of the target variable changes as a function of a specific lagged predictor, holding other variables constant. Second, SHAP values [27] were employed to decompose predictions into the contribution of each explanatory variable, allowing for the identification of both global importance and local effects.

This approach complements the Granger framework by not only assessing whether lagged relationships exist, but also by describing how and to what extent those relationships manifest across structurally distinct groups of economies.

## 5. Results

This section presents the empirical findings following the methodological pipeline described in Section 4. The results are organized according to the sequence of analysis: clustering analysis, correlation maps, Granger causality tests, and explanatory modeling.

### 5.1. Results on Cluster Analysis

Two clustering algorithms were evaluated, *k*-means and Agglomerative Clustering (both with k=2 to k=5), on the five annual macro-indicators (GDP growth, GDP per capita growth, inflation, unemployment, private credit). Both techniques achieved close Silhouette Scores for each *k*, as shown in Table 2.

Although the highest Silhouette Score was obtained for k=2, a two-cluster solution was considered overly restrictive to adequately represent heterogeneity. In particular, this specification tended to group together economies with substantially different macroeconomic profiles, such as more developed and financially deep countries alongside economies with intermediate structural characteristics or under-developed countries, resulting in clusters that were more non-intuitive in economic terms.

A comparison of the country composition for the k=2 and k=3 solutions in Table 3 further illustrates this limitation. It is worth noting that one of the clusters remains unchanged across both specifications, consistently grouping the most developed and stable economies; however, the remaining countries are aggregated into a single cluster under the k=2 specification. This masks relevant structural differences across these economies.

When k=3 is considered, this heterogeneous group is further disentangled into two distinct clusters, separating economies with intermediate structural characteristics from those with weaker macroeconomic conditions or lower levels of financial deepening. Importantly, this additional segmentation is achieved without significantly compromising the Silhouette Score, which remains at a reasonably high level (see Table 2). As a result, this specification provides a more granular and economically meaningful partition of the sample. Thus, this partition of clusters was selected, considering also that it preserves the stability of the most developed economies while improving the identification of structural differences among the remaining countries.

As an additional step, an analysis to show whether scaling the data would improve the clustering structure was performed. Several scaling techniques were tested, including standardization (z-score normalization), min–max scaling, robust scaling (based on median and interquartile range), and max-absolute scaling, and their performance was compared against the original unscaled data using both *k*-means and Agglomerative Clustering with k=3. The results, summarized in Table 4, show that the highest Silhouette Scores were consistently obtained when no scaling was applied. Although robust scaling yielded relatively close results, the remaining scaling methods led to a noticeable deterioration in cluster separation. This suggests that the original scale of the variables preserves economically meaningful differences across countries, which are partially distorted when normalization is imposed. Therefore, the clustering analysis was conducted using the non-scaled data.

**Figure 4:**
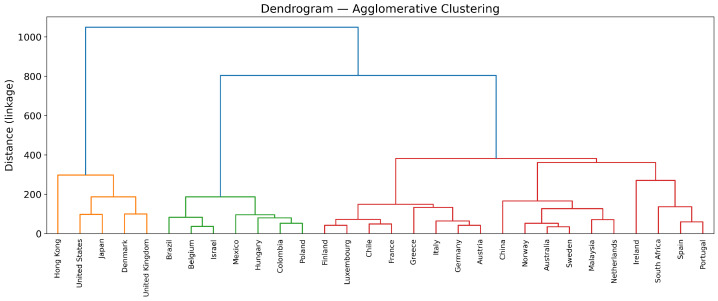
Ward-linkage dendrogram for the Agglomerative cluster solution. Colors indicate clusters.

**Figure 5:**
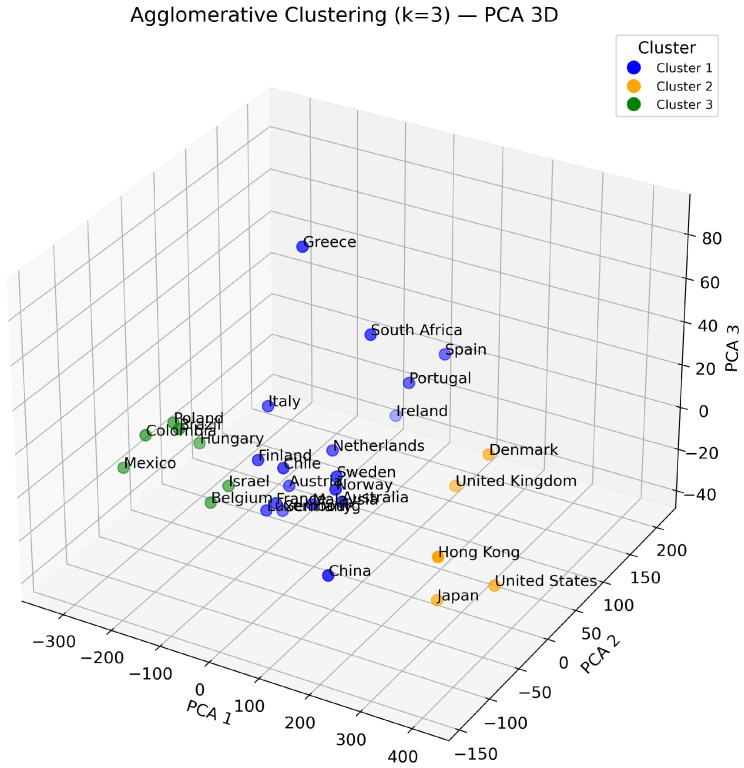
Agglomerative clustering visualized with 3D PCA.

Regarding the clustering algorithm, *k*-means produced acceptable partitions but with less clearly defined group boundaries. In contrast, Agglomerative Clustering generated more coherent and economically interpretable clusters. This was supported both by quantitative indicators and by visual inspection. The hierarchical structure revealed in the Ward-linkage dendrogram (Figure 4) illustrates the stability of the three-cluster solution, while the PCA projection in Figure 5 further confirms a well-separated segmentation. Consequently, Agglomerative Clustering was adopted as the preferred method.

**Figure 6:**
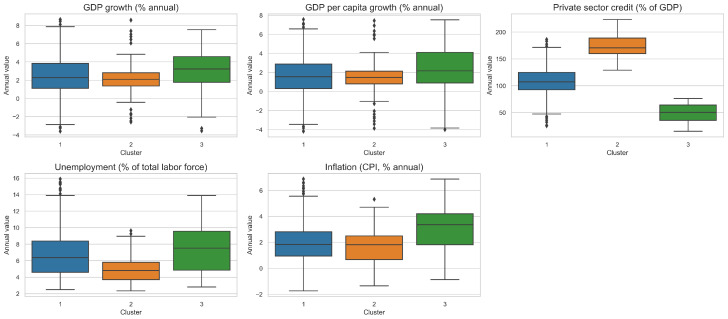
Distribution of macroeconomic indicators by cluster (outliers smoothed).

Figure 6 illustrates the distribution of macroeconomic indicators across clusters, where clear structural differences are notable. Cluster 1 represents countries with intermediate levels of private sector credit, and moderate unemployment and inflation; Cluster 2 displays a combination of contained GDP growth with the highest levels of private sector, and the lowest unemployment and inflation; finally, Cluster 3 shows the lowest financing, with the highest inflation and unemployment, with higher levels of GDP growth.

To improve the visual interpretability of the distributions, extreme outliers were filtered using the interquartile range (IQR) method. Specifically, for each variable, observations lying outside the interval [Q1−1.5·IQR,Q3+1.5·IQR] were excluded from the visualization. This smoothing approach was applied solely for graphical purposes and does not affect the underlying data used in the clustering procedure.

The clustering procedure identified three groups of countries with relatively homogeneous macroeconomic patterns within each cluster and clear differences across clusters.

Cluster 1, identified as “Developed and in transition economically stable”, comprises mainly developed economies together with countries in advanced transition. These economies are characterized by moderate growth rates, contained levels of inflation and unemployment, and intermediate financial depth.

Cluster 2, named as “Highly developed, competitive and stable”, includes the most consolidated economies in the sample. This group exhibits low inflation and unemployment, stable macroeconomic conditions, and high levels of private credit relative to GDP.

Finally, Cluster 3, labeled as “Emerging and dynamic intermediate markets with macroeconomic risk”, represents economies with stronger growth dynamics but also higher inflation and unemployment, combined with comparatively lower levels of financial deepening.

This structural segmentation provides the macroeconomic backdrop for the subsequent dynamic analysis.

To assess the robustness of the clustering structure, the analysis was repeated for the sub-period 2010–2019, excluding major global shocks such as the 2008–2009 Great Financial Crisis and the COVID-19 pandemic.

The results show that the group named “Highly developed, competitive and stable” is consistently identified as a distinct cluster, with almost identical membership in both periods (only grouping in another cluster the United Kingdom).

For the remaining countries, a number of reallocations are observed between clusters. When restricting the sample to the 2010–2019 sub-period, a larger set of economies is grouped within the cluster characterized as “Emerging and dynamic intermediate markets with macroeconomic risk”, with several countries previously classified under the “Developed and in transition economically stable” group being reassigned.

This pattern suggests that, under non-stressed conditions, certain intermediate economies exhibit macroeconomic dynamics that are closer to those of more developed countries, making the boundary between these groups less pronounced. In contrast, when considering the full sample, which includes periods of economic stress, these structural differences become more clearly distinguishable.

Overall, these results support the interpretation that the identified clusters reflect structural macroeconomic patterns rather than transient effects associated with specific time periods.

Notably, the Silhouette Score for the sub-period (0.4402) remains comparable to, and even slightly higher than, that of the full sample, reinforcing the stability of the clustering structure.

While the clustering structure remains stable when excluding major global shocks, the full sample (2005–2022) was retained for the main analysis. The rationale for this choice is that the broader time span allows for a more comprehensive characterization of the structural macroeconomic patterns of each country, incorporating different phases of the economic cycle. In particular, including both expansionary and contractionary periods provides a richer representation of the dynamics underlying financial development, growth, and macroeconomic stability.

Moreover, given that the clustering results are robust to the exclusion of crisis periods, as shown in Table 5, the use of the full sample does not appear to be driven by transient shocks, but rather reflects persistent structural differences across economies. Therefore, the baseline clustering based on the complete period is maintained for the subsequent analysis.

### 5.2. Results on Correlation Analysis

**Figure 7:**
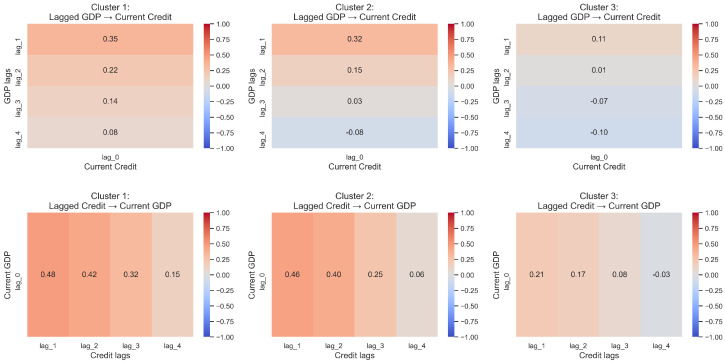
Correlation heatmaps by cluster. Top: Lagged GDP vs. current credit. Bottom: Lagged credit vs. current GDP.

Within each cluster, Pearson correlations between GDP growth and credit growth were computed considering contemporaneous and lagged relationships (up to four quarters). Then, for summarizing those patterns, heatmaps were constructed to visualize heterogeneous dynamics across groups, and are presented in Figure 7.

For all clusters, positive correlations are concentrated at short lags; however, the levels are lower for Cluster 3. These correlations suggest that movements in one variable tend to precede the other within a limited time window.

Although correlation does not imply causation, the presence of systematic lagged associations suggests potential predictive relationships. This also motivates formal directional tests. However, simple correlations suggest dynamic association but cannot establish direction nor control for own-lag persistence, hence the move to Granger tests.

### 5.3. Results on Granger Causality Tests

Before conducting the Granger causality tests, all series were transformed into year-over-year (YoY) growth rates in order to reduce non-stationarity and focus on short-term dynamics.

To assess whether time series are stationary, Augmented Dickey–Fuller (ADF) tests were performed for each country and variable. The results indicate that approximately 47% of the series are stationary at the 5% significance level for both GDP and credit.

While the transformation improves the statistical properties of the data, these results suggest that stationarity is not fully achieved in all cases. However, this limitation is common in macroeconomic time series and does not invalidate the use of Granger causality as an exploratory tool, particularly when the analysis focuses on relative patterns across structurally comparable groups rather than on precise parameter estimation. This is particularly relevant in macroeconomic applications, where strict stationarity is rarely fully satisfied in practice.

For each country, we performed Granger tests in both directions using quarterly lags p∈{1,2,3,4}, based on the models defined in Equations (5) and (6), and tested $H_{0}:\gamma_{1}=\cdots =\gamma_{p}=0$ with a *F*-test at the 5% significance level [23].

The results were summarized by cluster using the following rules: (i) *Unidirectional* if $H_{0}$ is rejected in α=0.05 in one direction only (at least one significant lag in one direction by the *F* test and none in the opposite direction); (ii) *Bidirectional* if $H_{0}$ is rejected at α=0.05 in both directions (at least one significant lag in both directions); and (iii) *No evidence* if $H_{0}$ is not rejected at α=0.05 in either direction.

According to the Table 6, it is clear that Cluster 1, “Developed and in transition economically stable”, concentrates on *Credit → GDP* causality. Cluster 2, “Highly developed, competitive and stable” countries, is mostly focused on bidirectional causality. While, finally, Cluster 3, of “Emerging and dynamic intermediate markets with macroeconomic risk”, exhibits mixed patterns with frequent bidirectionality, with a little bias to *Credit → GDP* causality, for which it is more difficult to reach a single direction or rule of causality.

To highlight the value added by the clustering-conditioned approach, a pooled Granger causality analysis was conducted across all countries, without accounting for structural heterogeneity. The results, summarized in Table 7, reveal a dispersed and less conclusive pattern. A substantial number of countries exhibit bidirectional causality (11 cases), while others display conflicting unidirectional relationships, with 14 countries showing *Credit → GDP* causality and only 2 exhibiting *GDP → Credit* causality. Additionally, three countries present no statistically significant causal relationship.

This distribution suggests that, when all economies are analyzed jointly, the resulting patterns become heterogeneous and difficult to interpret in a unified framework. In contrast, the clustering-conditioned analysis yields more coherent and economically meaningful relationships within structurally similar groups.

These findings highlight that ignoring macroeconomic heterogeneity may obscure the underlying finance–growth dynamics, reinforcing the relevance of the proposed segmentation approach. In addition, the predominance of bidirectional causality in a non-segmented dataset suggests that aggregated analyses may mask directional effects that only become apparent when structural differences across economies are taken into account.

However, Granger causality identifies directionality but does not quantify the magnitude or functional form of the effects. For this reason, we proceed to explanatory modeling.

### 5.4. Results on Explanatory Models

#### 5.4.1. Lasso-Based Lag Selection

Since part of the objective was not only to identify the direction of causality but also the magnitude and, especially, the timing of the effect (i.e., how many quarters it takes for one variable to impact the other, and which ones), Lasso regression was first applied within each cluster and for each causal direction to detect the most representative lags based on the magnitude of the coefficients.

Because quarterly lags of the same variable tend to be highly correlated, Lasso works here as a practical selection mechanism: it shrinks less informative coefficients toward zero and keeps only the lags that truly add explanatory value.

Table 8 summarizes the representative lags selected by Lasso within each structural cluster and causal direction. The results confirm that the temporal transmission mechanism differs across groups.

In Cluster 1, only the direction from credit to GDP remains active, with the first and second quarterly lags selected, suggesting a short but slightly persistent transmission effect. In Clusters 2 and 3, the relationship appears more immediate, as only the first lag is retained in both directions.

The results show that the effect is not evenly distributed across lags. In several clusters, only one or two specific quarters remain active, suggesting that the temporal transmission mechanism differs structurally across types of economies. These selected lags were then used as inputs in the subsequent non-linear modeling stage.

#### 5.4.2. XGBoost and Non-Linear Effects

After identifying the representative lag structure with Lasso, non-linear models were adjusted using XGBoost for each cluster and causal direction applicable. The objective of this stage is primarily interpretative: we aim to characterize the shape, intensity, and potential thresholds of the lagged effects, rather than to optimize out-of-sample forecasting performance.

Across all estimated cases, the XGBoost models achieved very high in-sample fit (with adjusted $R^{2}$ values consistently close to 1). This is expected given the flexibility of tree-based boosting methods and the relatively low-dimensional input space defined by the selected lags by Lasso.

It is important to notice that, in this setting, the main objective is not predictive performance or generalization, but rather to leverage the interpretability of the fitted models. In particular, the analysis focuses on Partial Dependence Plots (PDPs) and SHAP values, which allow us to summarize the functional form of the effect and the relative contribution of each selected lag.

While the high in-sample fit may suggest potential overfitting, this does not invalidate the interpretability analysis for two main reasons. First, the models are estimated on a reduced set of predictors selected through Lasso, which limits model complexity. Second, XGBoost incorporates regularization mechanisms that constrain the learning process. As a result, the extracted PDP and SHAP patterns were found to be stable and consistent across specifications. Therefore, the purpose of this modeling step is not to assess out-of-sample predictive performance, but to provide a structured interpretation of the marginal effects and relative importance of the lagged variables.

##### Cluster 1: Credit (Lags 1–2) → GDP

Granger tests pointed to predominantly causality from credit to GDP in this group, and Lasso retained two lagged terms of credit growth (t−1 and t−2) as relevant explanatory inputs for GDP growth.

**Figure 8:**
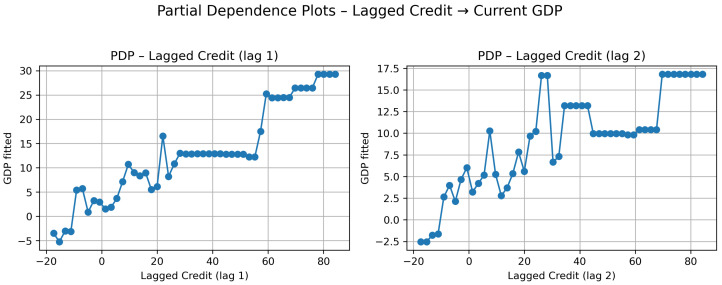
Cluster 1: Partial Dependence Plots for lagged credit.

The Partial Dependence Plots (Figure 8) reveal a non-linear transmission mechanism from credit growth to GDP growth in this group.

For the first lag (t−1), strongly negative credit growth (below approximately −10%) is associated with clearly negative GDP predictions. In this region, contractions in credit translate almost directly into economic slowdown. As credit growth moves into moderate positive territory (roughly between 0% and 20%), the slope indicates that increases in credit conduct proportional increases in GDP.

However, between approximately 25% and 50% credit growth, the curve flattens. In this interval, additional expansions in credit produce only marginal changes in GDP. This suggests a temporary saturation effect: once credit growth reaches moderate-high levels, further acceleration does not immediately amplify economic growth.

Beyond roughly 55% credit growth, a second upward shift appears. GDP predictions increase sharply again, reaching substantially higher levels. This indicates a threshold effect, where unusually strong credit expansions are associated with disproportionately large economic responses. In other words, moderate credit growth stabilizes output, but extreme expansions may trigger a stronger acceleration phase.

For the second lag (t−2), the same qualitative pattern emerges but with lower intensity. Negative credit still implies negative GDP effects, and moderate positive credit growth increases GDP. Nevertheless, the plateau region is more pronounced and the second upward shift is weaker compared to the first lag. This confirms that the transmission is primarily immediate, with diminishing strength in the second quarter.

**Figure 9:**
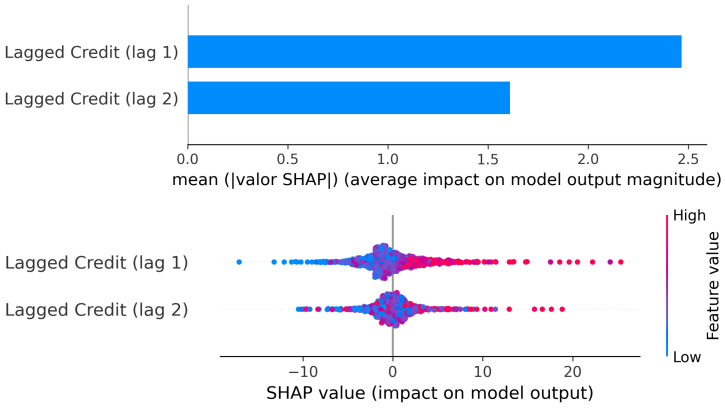
Cluster 1: SHAP values: Lagged Credit → Current GDP.

The SHAP values (Figure 9) reinforce this interpretation. The mean absolute SHAP magnitude is clearly higher for lag 1 than for lag 2, indicating that the most recent credit growth carries greater explanatory weight in the model. The SHAP scatter also shows that high positive credit values (red points) are associated with strong positive contributions to GDP predictions, while large negative credit values (blue points) contribute negatively.

Importantly, around moderate credit values (near zero to moderately positive), SHAP contributions cluster closer to zero, consistent with the plateau region observed in the PDP. This confirms that not all increases in credit generate equally strong GDP responses; the marginal effect depends on the level of credit growth.

Overall, the evidence suggests that in this cluster, credit acts as an active driver of growth, but the effect is non-linear: it is strong when recovering from contractions, stabilizes at intermediate expansion levels, and intensifies again under exceptionally large credit booms.

##### Cluster 2: GDP (Lag 1) → Credit

In this group and all the next ones, the Granger tests suggest bidirectionality, and Lasso retained only the first lag of GDP growth (t−1). This already indicates that, if there is transmission, it is short-run and mostly immediate.

**Figure 10:**
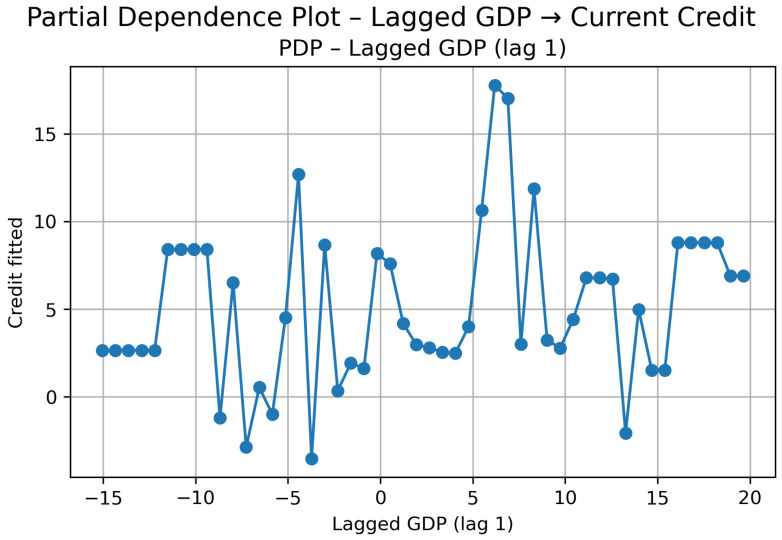
Cluster 2: PDP—Lagged GDP (lag t−1) → Current Credit.

The Partial Dependence Plot (Figure 10) shows that the effect is not cleanly increasing nor fully stable—it moves in segments.

For strongly negative GDP growth (around −15% to −8%), predicted credit growth remains mostly positive and relatively stable. This suggests that even during sharp contractions, credit does not collapse proportionally; the relationship is not symmetric in downturns.

Between roughly −7% and 0%, the behavior becomes more irregular. There is noticeable volatility in predicted credit, indicating that in mild recessions the response of credit is unstable rather than monotonic.

Once GDP growth turns moderately positive (around 0% to 8%), credit predictions increase and even reach a clear peak in the mid-positive range. However, this increase is not persistent. After this local maximum, the curve stabilizes again.

At higher GDP growth levels (above approximately 15%), the PDP becomes flatter. Credit growth concentrates in a narrower band, suggesting that stronger economic growth does not translate into proportionally higher credit expansion. In other words, there is no explosive amplification effect; the response seems bounded.

**Figure 11:**
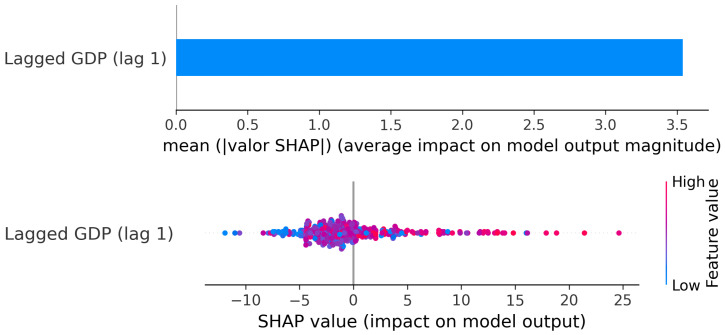
Cluster 2: SHAP values: Lagged GDP (lag t−1) → Current Credit.

The SHAP results (Figure 11) reinforce this reading. Since only one lag is included, the mean absolute SHAP value reflects the overall relevance of GDP_*t*−1_ in the model. The dispersion plot shows that most observations cluster around small SHAP values, meaning that in many cases GDP adds limited marginal contribution to credit predictions.

The strongest positive SHAP contributions are associated with high positive GDP growth (red points on the right). By contrast, negative GDP growth values (blue points) tend to generate weaker and more dispersed effects. This asymmetry is consistent with the PDP: strong expansions can push credit upward, but contractions do not generate an equally strong and systematic decline.

Overall, in this cluster, the GDP → credit channel exists at a short horizon, but it is not smooth nor strictly proportional. The effect appears in specific growth regimes rather than as a persistent or dominant transmission mechanism.

##### Cluster 2: Credit (Lag 1) → GDP

**Figure 12:**
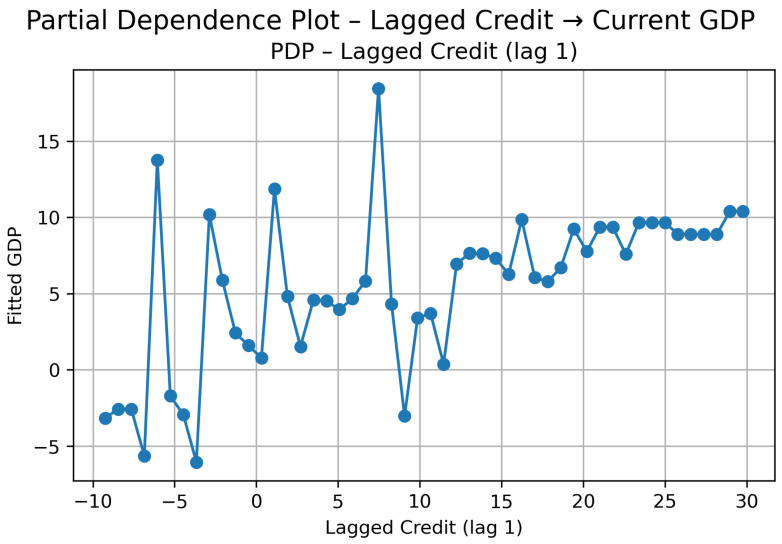
Cluster 2: PDP—Lagged Credit (lag t−1) → Current GDP.

The PDP for Cluster 2 (Figure 12) shows a clearly positive but smoother relationship compared to Cluster 1.

For negative credit growth (below approximately −5%), GDP predictions remain low and relatively flat, indicating that contractions in credit are associated with weaker economic performance, although the decline is not explosive. As credit growth moves from slightly negative to moderately positive values (roughly between 0% and 10%), GDP increases steadily. In this region, the slope is positive and credit expansions translate into proportional increases in output.

Between approximately 10% and 20%, the curve becomes smoother and slightly less steep. The effect remains positive, but the marginal impact begins to moderate. Unlike Cluster 1, there is no sharp second acceleration phase; instead, GDP stabilizes around higher levels and grows more gradually as credit continues to expand.

Overall, the pattern suggests a relatively smoother transmission compared to Cluster 1. Credit growth supports GDP growth in a broadly positive way, but the effect is gradual and bounded. There are no sharp threshold jumps, and the marginal impact tends to moderate as credit expands further. The relationship is present, but not dominant.

**Figure 13:**
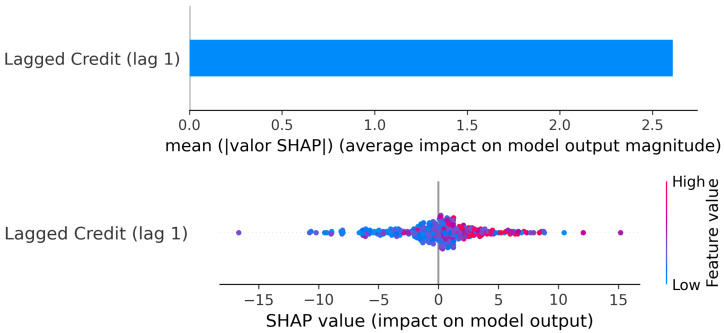
Cluster 2: SHAP values: Lagged Credit (lag t−1) → Current GDP.

The SHAP results (Figure 13) confirm this interpretation.

The mean absolute SHAP value indicates that lagged credit carries substantial explanatory weight in the model.

It is worth noting that, in this cluster, the SHAP dispersion is narrower than in Cluster 1. This reinforces the idea that, in highly developed and stable economies, credit expansions affect GDP in a more controlled and linear-like manner. There is evidence of diminishing marginal impact at higher levels of credit growth, but no abrupt regime shift.

In short, for Cluster 2, credit still leads GDP, but the mechanism appears smoother and more proportional than in the previous group.

Despite the fact that both directions are statistically significant according to the Granger tests and are supported by the non-linear models, the overall pattern in Cluster 2 is less structurally clear than in Cluster 1. In neither direction does the relationship display a clean or dominant transmission mechanism. The PDPs show fluctuations, local thresholds, and saturation regions, but not a channel where one variable consistently drives the other.

This suggests that, although predictive interaction exists, neither GDP nor credit appears to depend heavily on the other in a systematic way within this group. One possible interpretation is that these are highly developed and competitive economies where both output and credit levels are structurally high and relatively stable. In such environments, financial deepening is already mature and economic growth may be driven by multiple complex factors beyond short-term credit dynamics. As a result, the finance–growth nexus exists, but it operates in a more contained and balanced manner compared to the more credit-driven structure observed in Cluster 1.

This pattern is consistent with the theoretical literature from Arcand et al. [28] on financial development, which suggests that in mature and highly developed economies, the marginal contribution of credit to growth tends to diminish as financial systems become deeper and more efficient. In such contexts, the finance–growth relationship is often mediated by more complex structural factors, and becomes less dependent on short-term fluctuations in credit volumes.

##### Cluster 3: GDP (Lag 1) → Credit

**Figure 14:**
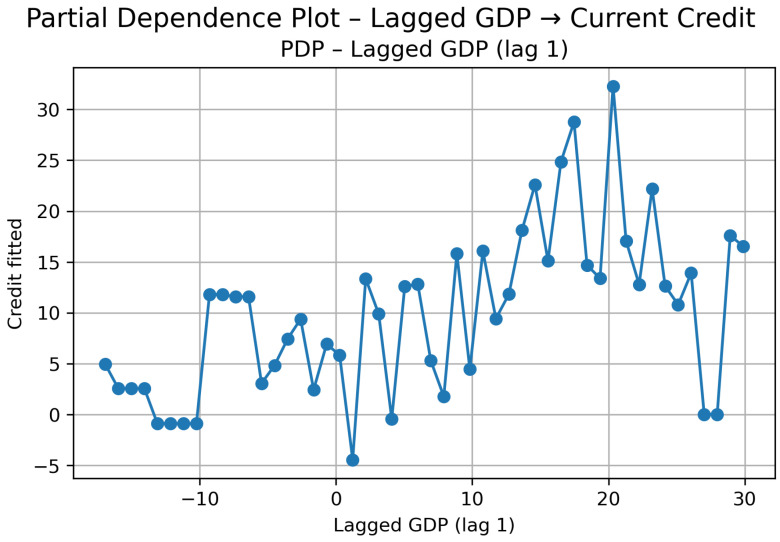
Cluster 3: PDP—Lagged GDP (lag t−1) → Current Credit.

For Cluster 3, unlike Cluster 2, the PDP here shows stronger non-linearities and more visible regime shifts, as shown in Figure 14.

For strongly negative GDP growth (below approximately −10%), predicted credit growth remains low and relatively flat, even slightly negative in some points. This suggests that in severe contractions, credit expansion is clearly constrained, but not collapsing dramatically. The response is contained rather than explosive.

Between roughly −10% and 0%, the curve becomes more irregular. Credit predictions fluctuate noticeably, indicating that during mild recessions or weak growth episodes, the reaction of credit is unstable and highly state-dependent.

Once GDP growth turns positive (around 0% to 10%), the slope becomes clearly increasing. In this region, improvements in economic activity translate into visibly higher credit growth. The relationship is much more interpretable here than in negative territory.

Between approximately 10% and 20%, the curve continues rising and even reaches a local maximum above 20% predicted credit growth. This indicates a strong amplification phase: in high-growth episodes, credit expansion accelerates significantly.

However, beyond that peak (around 20% GDP growth and above), the curve does not continue rising proportionally. Credit predictions begin to fluctuate and even decline slightly at the highest GDP values, suggesting a saturation or ceiling effect. In other words, extremely high growth does not indefinitely amplify credit.

**Figure 15:**
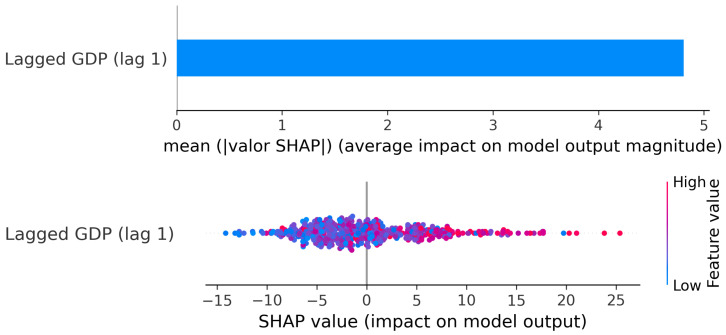
Cluster 3: SHAP values: Lagged GDP (lag t−1) → Current Credit.

The SHAP results (Figure 15) reinforce this interpretation.

The mean absolute SHAP value is relatively large, indicating that GDP_*t*−1_ carries substantial explanatory weight in this group. The dispersion plot shows that high positive GDP growth (red points on the right) generates strong positive SHAP contributions, pushing credit predictions upward. In contrast, negative GDP values (blue points) tend to produce moderate but not extreme negative contributions.

Overall, in Cluster 3, the credit appears more reactive and more dependent on GDP changes than in Cluster 2. Credit responds clearly during expansion phases, especially in high-growth environments, but the response is bounded and does not grow indefinitely. This pattern is consistent with emerging or intermediate economies, where credit cycles tend to amplify growth phases but remain constrained by macroeconomic risk and structural limits.

##### Cluster 3: Credit → GDP (Lag 1)

In Cluster 3, the transmission from credit to GDP appears economically meaningful and more clearly structured than in Cluster 2, although it remains non-linear.

**Figure 16:**
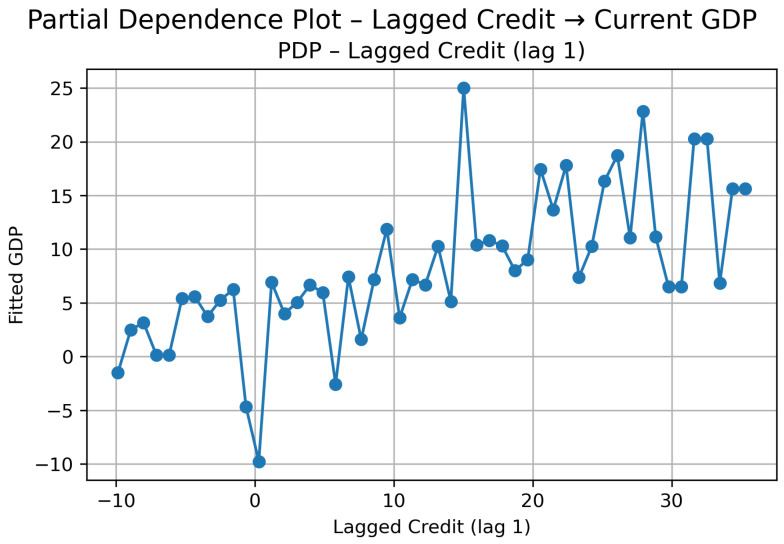
Cluster 3: PDP—Lagged Credit (lag t−1) → Current GDP.

The PDP (Figure 16) shows that when credit growth is negative (below roughly −5%), predicted GDP growth declines noticeably. Around zero or slightly negative credit growth, GDP reaches some of its lowest predicted levels. This suggests that weak or stagnant credit conditions are closely associated with economic slowdown in this group.

As credit growth turns positive (approximately between 0% and 15%), GDP increases in a relatively steady and upward-sloping manner. In this range, the relationship is clearly positive and fairly monotonic: moderate credit expansions translate into higher output growth.

Between roughly 15% and 25%, GDP continues increasing, but the marginal impact becomes smaller. This indicates a gradual saturation effect: credit still supports growth, but each additional increase contributes less than before.

At very high credit growth levels (above around 25%), the curve becomes more irregular. GDP fluctuates within a higher range rather than accelerating continuously. This suggests that extreme credit booms do not produce proportionally stronger output gains, reinforcing the idea of diminishing marginal returns.

**Figure 17:**
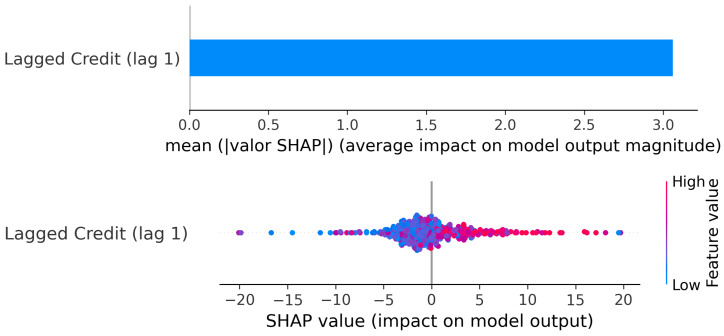
Cluster 3: SHAP values: Lagged Credit (lag t−1) → Current GDP.

The SHAP results (Figure 17) confirm the strength of this channel. The mean absolute SHAP value is relatively high, indicating that lagged credit has substantial explanatory weight in the model.

Overall, in Cluster 3, credit behaves as an active short-run driver of GDP, particularly in the transition from weak to moderate expansion phases. However, the effect is clearly non-linear and bounded: once credit growth becomes very large, the marginal impact on GDP stabilizes rather than accelerating indefinitely.

Taking both directions into account, Cluster 3 displays a more coherent dynamic interaction than Cluster 2, which also resulted in bidirectional feedback.

In the GDP → credit direction, the PDP suggests that moderate and strong positive GDP growth is associated with clear increases in credit expansion, while severe negative GDP episodes do not generate a proportionally large contraction in credit. Credit appears to remain relatively contained during downturns and reacts more visibly once economic activity stabilizes and turns positive. This may reflect that, in these economies, credit expansion requires a minimum level of macroeconomic certainty before accelerating.

In the credit → GDP direction, the PDP shows a predominantly positive and more monotonic pattern. Once credit growth turns positive and gains momentum, GDP responds in the same direction, with increasingly higher predicted values. Negative credit growth is associated with weaker output, but the strongest responses are observed in moderate-to-high positive credit ranges.

Taken together, this suggests a feedback mechanism rather than one-sided dominance. Economic stabilization and moderate growth appear to enable credit expansion, and once credit expands, it further supports output growth in subsequent quarters. At the same time, neither direction behaves explosively under extreme negative values, indicating that the interaction is strong but not mechanically proportional.

In contrast to Cluster 2, where the relationship appeared diffuse and less structured, Cluster 3 shows a more economically intuitive cycle: macroeconomic improvement supports financial expansion, and financial expansion feeds back into growth. This reinforces the idea that in emerging and intermediate markets with macroeconomic risk, the finance–growth nexus operates as a dynamic reinforcement process rather than as a smooth, fully mature transmission channel.

## 6. Discussion

### 6.1. Synthesis of Findings

The results obtained across clustering, Granger causality analysis, and non-linear modeling, confirm that the finance–growth nexus is structurally heterogeneous and cannot be summarized by a single universal pattern.

The clustering stage was not merely a descriptive segmentation exercise; it materially changed the interpretation of the dynamic results. Once countries were grouped according to macroeconomic structure, the direction and intensity of the predictive relationships between credit and GDP differed in a consistent way. This reinforces the idea that the finance–growth relationship is conditional on structural characteristics such as financial depth, macroeconomic stability, and development stage.

In Cluster 1 (developed and in transition economically stable), the dominant pattern is credit → GDP. Both Granger tests and non-linear modeling suggest that credit expansions precede output growth, with short-run persistence (lags 1 and 2). Importantly, the XGBoost stage revealed that this transmission is non-linear: moderate expansions stabilize output, while very strong credit growth can generate acceleration phases. This suggests that in these economies, financial conditions play an active role in shaping short-term growth dynamics.

Cluster 2 (Highly developed, competitive and stable) presents a different configuration. Although statistical bidirectionality appears in the Granger framework, the non-linear results do not reveal a dominant or structurally clean transmission channel. Both directions exist, but neither displays strong threshold effects or explosive dynamics. The relationships are smoother, bounded, and less dependent on specific regimes. This may reflect mature financial systems where both credit and output operate at structurally high and stable levels, and where growth depends on a broader set of factors beyond short-run credit fluctuations. In this context, the finance–growth nexus appears more balanced and less dominant.

Cluster 3 (emerging and dynamic intermediate markets with macroeconomic risk) exhibits a more interpretable feedback mechanism. GDP improvements are followed by credit acceleration, and credit expansion subsequently supports GDP growth. The relationship is clearly non-linear and state-dependent: positive growth phases amplify financial deepening, while extreme values do not generate proportional responses. Compared to Cluster 2, the interaction here is more structured and economically intuitive, resembling a reinforcement cycle between macroeconomic stabilization and financial expansion. At the same time, the bounded behavior at extreme values suggests the presence of structural constraints and macroeconomic risk.

Taken together, these findings indicate that the direction and strength of predictive dynamics depend on the stage of development and macroeconomic configuration. In some groups, credit acts as a leading driver; in others, the relationship is balanced and diffuse; and in intermediate economies, a feedback mechanism emerges.

### 6.2. Implications for the Finance–Growth Debate

From a broader perspective, the results provide empirical nuance to the classical debate initiated by early contributions such as [1,2,9] and later expanded in [3,4,5]. Rather than supporting a single dominant hypothesis (finance-led growth or growth-led finance), the evidence suggests that both mechanisms may coexist, but their relative importance depends on structural conditions.

The heterogeneity observed across clusters aligns with the idea that financial deepening does not operate uniformly across countries. In financially mature economies, marginal credit expansions may have limited incremental impact on growth. In contrast, in developing or intermediate economies, credit can amplify growth cycles more visibly, although still within bounded ranges.

Additionally, the non-linear patterns detected through PDP and SHAP analysis suggest that the relationship is rarely proportional. Threshold effects, saturation regions, and diminishing marginal impacts appear across clusters. This indicates that the finance–growth nexus may be better characterized as state-dependent rather than linear.

### 6.3. Model Limitations

Despite the insights obtained from the presented analyses, a few limitations should be acknowledged. First, the study relies on aggregate private sector credit, and while this facilitates comparability across countries, it might hide relevant differences within the financial system. In particular, household and corporate credit often behave differently and may have distinct effects on economic activity. Therefore, the results should be interpreted as capturing broad financial development dynamics rather than more granular credit mechanisms.

Second, although the clustering stage includes key macroeconomic indicators, it does not explicitly incorporate institutional factors such as regulatory quality, governance, or legal factors. These elements might be important drivers of the finance–growth relationship and could influence both the direction and strength of the observed interactions. As a result, part of the cross-country heterogeneity may not be fully captured in the current specification.

These limitations open several natural extensions. A more detailed analysis distinguishing between types of credit could help uncover more specific transmission channels. Similarly, incorporating institutional and fiscal policy variables into the clustering stage may lead to a more refined segmentation and clearer economic interpretation. More generally, combining macroeconomic and institutional dimensions in the segmentation process appears to be a promising direction to further understand the finance–growth nexus.

## 7. Conclusions

This analysis studied the nexus of finance and economic growth under a structural heterogeneity framework. Rather than assuming a universal direction of causality, countries were first segmented according to their macroeconomic profiles and only then were dynamic interactions evaluated within each group.

Four main takeaways emerge: first, the causality of each variable on the other depends on the structural conditions of the economies. Second, the relation between both variables is non-linear; further, there are cases when the effect of one on the other is marginal or without impact. Third, the greater the dependency of the two variables, the less developed countries are. The statistical results show causality; however, the difficulty to interpret the results intuitively shows that, for the most developed and competitive countries, the relationship might be more complex and needs to take into account other factors.

Overall, the findings suggest that the studied relationship should not be framed as a single theoretical dispute (finance-led versus growth-led), but as a context-dependent mechanism shaped by macroeconomic structure.

It becomes clearer that the contrast of theories analyzed does not result in a single winner, and both the starting authors’ (Schumpeter and Robinson) approaches are finalized to be partially correct—or incorrect—depending on the reader’s ideology.

With these findings, it becomes clear that the classical finance–growth debate does not yield a single winner. In different structural environments, both Schumpeter’s finance-led hypothesis and Robinson’s growth-led argument find empirical support. Whether one appears “correct” may depend less on ideology and more on the macroeconomic structure under analysis.

Future research may extend this framework by incorporating institutional variables, fiscal indicators, or longer historical windows.

## Figures and Tables

**Table 1:** Descriptive statistics of main variables in our dataset.

Variable	Min	Mean	StdDev	Max
GDP growth (%)	−10.94	2.27	3.64	24.62
Per Capita GDP Growth (%)	−11.37	1.56	3.58	23.44
Inflation (%)	−4.45	2.52	2.27	14.61
Unemployment	2.35	7.89	5.25	34.01
Private lending (% of GDP)	15.31	106.36	47.12	262.44

**Table 2:** Silhouette Scores obtained by the two clustering algorithms (*k* = 2–5).

*k*	Agglomerative	*k*-Means
2	0.4506	0.4237
3	0.4081	0.4048
4	0.3274	0.3622
5	0.3522	0.3667

**Table 3:** Comparison of country composition for k=2 and k=3 cluster solutions.

Specification	Cluster	Countries
k=2	Cluster 0	Germany, Australia, Austria, Brazil, Belgium, Chile, China, Colombia, Spain, Finland, France, Greece, Hungary, Ireland, Israel, Italy, Luxembourg, Malaysia, Mexico, Norway, The Netherlands, Poland, Portugal, South Africa, Sweden
	Cluster 1	Denmark, United States, Hong Kong SAR, Japan, United Kingdom
k=3	Cluster 0	Germany, Australia, Austria, Chile, China, Spain, Finland, France, Greece, Ireland, Italy, Luxembourg, Malaysia, Norway, The Netherlands, Portugal, South Africa, Sweden
	Cluster 1	Denmark, United States, Hong Kong SAR, Japan, United Kingdom
	Cluster 2	Brazil, Belgium, Colombia, Hungary, Israel, Mexico, Poland

**Table 4:** Silhouette Scores obtained with different scaling methods using *k*-means and Agglomerative Clustering (*k* = 3).

Scaler	Agglomerative	*k*-Means
No scaling	0.4081	0.4048
RobustScaler	0.3797	0.3797
MaxAbsScaler	0.2426	0.1597
StandardScaler	0.1507	0.2277
MinMaxScaler	0.1509	0.1733

**Table 5:** Comparison of cluster composition between full sample (2005–2022) and sub-period (2010–2019).

Period	Cluster	Countries
2005–2022	Cluster 0	Germany, Australia, Austria, Chile, China, Spain, Finland, France, Greece, Ireland, Italy, Luxembourg, Malaysia, Norway, The Netherlands, Portugal, South Africa, Sweden
	Cluster 1	Denmark, United States, Hong Kong SAR, Japan, United Kingdom
	Cluster 2	Brazil, Belgium, Colombia, Hungary, Israel, Mexico, Poland
2010–2019	Cluster 0	Germany, Austria, Brazil, Belgium, Colombia, Finland, France, Hungary, Ireland, Israel, Italy, Luxembourg, Mexico, Poland
	Cluster 1	Australia, Chile, China, Spain, Greece, Malaysia, Norway, The Netherlands, Portugal, United Kingdom, South Africa, Sweden
	Cluster 2	Denmark, United States, Hong Kong SAR, Japan

**Table 6:** Granger causality outcomes by cluster (number of countries).

Cluster	Bidirectional	Credit → GDP	GDP → Credit	No Evidence
**1**	4	11	2	1
**2**	4	1	0	0
**3**	3	2	0	2

Notes: Cluster 1 = Developed and in transition economically stable. Cluster 2 = Highly developed, competitive and stable. Cluster 3 = Emerging and dynamic intermediate markets with macroeconomic risk.

**Table 7:** Distribution of Granger causality patterns in the pooled sample.

Causality Pattern	Number of Countries
Bidirectional	11
Credit → GDP	14
GDP → Credit	2
No causality	3

**Table 8:** Representative lags selected by Lasso within each cluster and causal direction.

Cluster	Direction	Selected Lags
1	Credit → GDP	t−1, t−2
2	GDP → Credit	t−1
2	Credit → GDP	t−1
3	GDP → Credit	t−1
3	Credit → GDP	t−1

Notes: Cluster 1 = Developed and in transition economically stable. Cluster 2 = Highly developed, competitive and stable. Cluster 3 = Emerging and dynamic intermediate markets with macroeconomic risk.

## Data Availability

The data used in this study are publicly available from international institutions. Quarterly GDP data were obtained from the International Monetary Fund (IMF), *Quarterly National Accounts*, available at https://data.imf.org/ (accessed on 23 March 2025); data on credit to the private non-financial sector were retrieved from the Bank for International Settlements (BIS), *Total Credit Statistics*, available at https://www.bis.org/statistics/totcredit.htm (accessed on 23 March 2025); macroeconomic structural indicators (GDP growth, GDP per capita growth, inflation, and unemployment) were collected from the World Bank, *World Development Indicators (WDI)*, available at https://databank.worldbank.org/source/world-development-indicators (accessed on 23 March 2025). No new primary data were generated for this study. All datasets are publicly accessible and were processed and combined by the authors for analytical purposes.

## Abbreviations

The following abbreviations are used in this manuscript:

GDP	Gross Domestic Product
BIS	Bank for International Settlements
IMF	International Monetary Fund
WDI	World Development Indicators
PCA	Principal Component Analysis
DTW	Dynamic Time Warping
OLS	Ordinary Least Squares
PDP	Partial Dependence Plot
SHAP	SHapley Additive exPlanations
